# Comparison of choose-a-movie and approach–avoidance paradigms to measure social motivation

**DOI:** 10.1007/s11031-017-9647-1

**Published:** 2017-11-11

**Authors:** Indu Dubey, Danielle Ropar, Antonia Hamilton

**Affiliations:** 1 School of Psychology & Clinical Language Sciences, University of Reading, Harry Pitt Building, Earley Gate, Reading, RG6 6AL UK; 2 School of Psychology, University of Nottingham, University Park, Nottingham, NG7 2RD UK; 3 Institute of Cognitive Neuroscience, University College London, Alexandra House, 17 Queen Square, London, WC1N 3AR UK

**Keywords:** Approach/avoidance, Choose-a-movie (CAM), Social seeking, Social motivation, Autistic traits

## Abstract

Social motivation is a subjective state which is rather difficult to quantify. It has sometimes been conceptualised as “behavioural effort” to seek social contact. Two paradigms: approach–avoidance (AA) and choose a movie (CAM), based on the same conceptualisation, have been used to measure social motivation in people with and without autism. However, in absence of a direct comparison, it is hard to know which of these paradigms has higher sensitivity in estimating preference for social over non-social stimuli. Here we compare these two tasks for their utility in (1) evaluating social seeking in typical people and (2) identifying the influence of autistic traits on social motivation. Our results suggest that CAM reveals a clear preference for social stimuli over non-social in typical adults but AA fails to do so. Also, social seeking measured with CAM but not AA has a negative relationship between autistic traits.

## Introduction

Motivation has been defined as a drive to reduce the negative outcome or increase the positive outcome by acting on the environment (Hill 1963), as a biological mechanism to maintain the homeostasis (Hull 1943), or as an adjustment of opponent processes (Solomon 1980). In most of these definitions, motivation is seen as an internal state which results in behavioural activation. This conceptualisation of motivation is not limited to basic physiological drives but is also extended to social situations. Social motivation is defined as “a set of psychological dispositions and biological mechanisms biasing the individual to preferentially orient to the social world (social orienting), to seek and take pleasure in social interactions (social reward), and to work to foster and maintain social bonds (social maintaining)” (Chevallier et al. 2012, p. 2), as this definition suggests social interactions are inherently rewarding for typical people, therefore they make an effort to seek social contacts. This means that the “behavioural effort” or “seeking actions” of the person can give an estimate of his/her subjective state of motivation.

Based on this assumption, researchers have developed behavioural paradigms that estimate the strength of approach motivation by measuring effort made by the person. Here we compare two paradigms the approach–avoidance (AA) paradigm and the choose a movie (CAM) paradigm for their sensitivity to measure social seeking opposed to non-social stimuli. The AA paradigms generally involve presentation of stimuli images on a computer screen while participants are encouraged to press a button on a computer keyboard to increase/decrease the duration of presentation of the images (Aharon et al. 2001). To make the key presses effortful, some researchers have used a combination of difficult key presses such as a two-button sequence using the same finger (Aharon et al. 2001; Ewing et al. 2013). The other form of AA paradigms involves pulling (approaching) or pushing (avoiding) the joystick to change the size of the stimulus image (Heuer et al. 2007; Silva et al. 2015). AA paradigms have been used to measure food approach–avoidance bias (Paslakis et al. 2016), avoidance motivation for fear of spiders (Rinck and Becker 2007), approach motivation for attractive and non-attractive images of people (Hayden et al. 2007), social / non-social stimuli seeking behaviour (Ewing et al. 2013).

However, there are some limitations of AA paradigm as it is typically used in the previous studies, first, in all these AA paradigms the stimulus under investigation is presented as a static image which is not as ecologically valid as dynamic stimuli. Second, in AA paradigms the stimulus is fully visible to the participants when they make the decision to either approach or avoid it. This makes it hard to distinguish if the participants’ decision about approaching a stimulus has emerged from his/her high motivation to look at that specific image, perhaps due to its colour/contrast/style or if he/she has a high approach motivation for the category this stimulus belongs to such as faces or cars etc. Third, in AA paradigms participants have no alternative stimulus that they can choose to view, which is unlike a real life situation where we always have multiple options to choose from. All these limitations might influence the sensitivity of this measure, making it hard to generalise its results to real life social seeking behaviour.

**Fig. 1:**
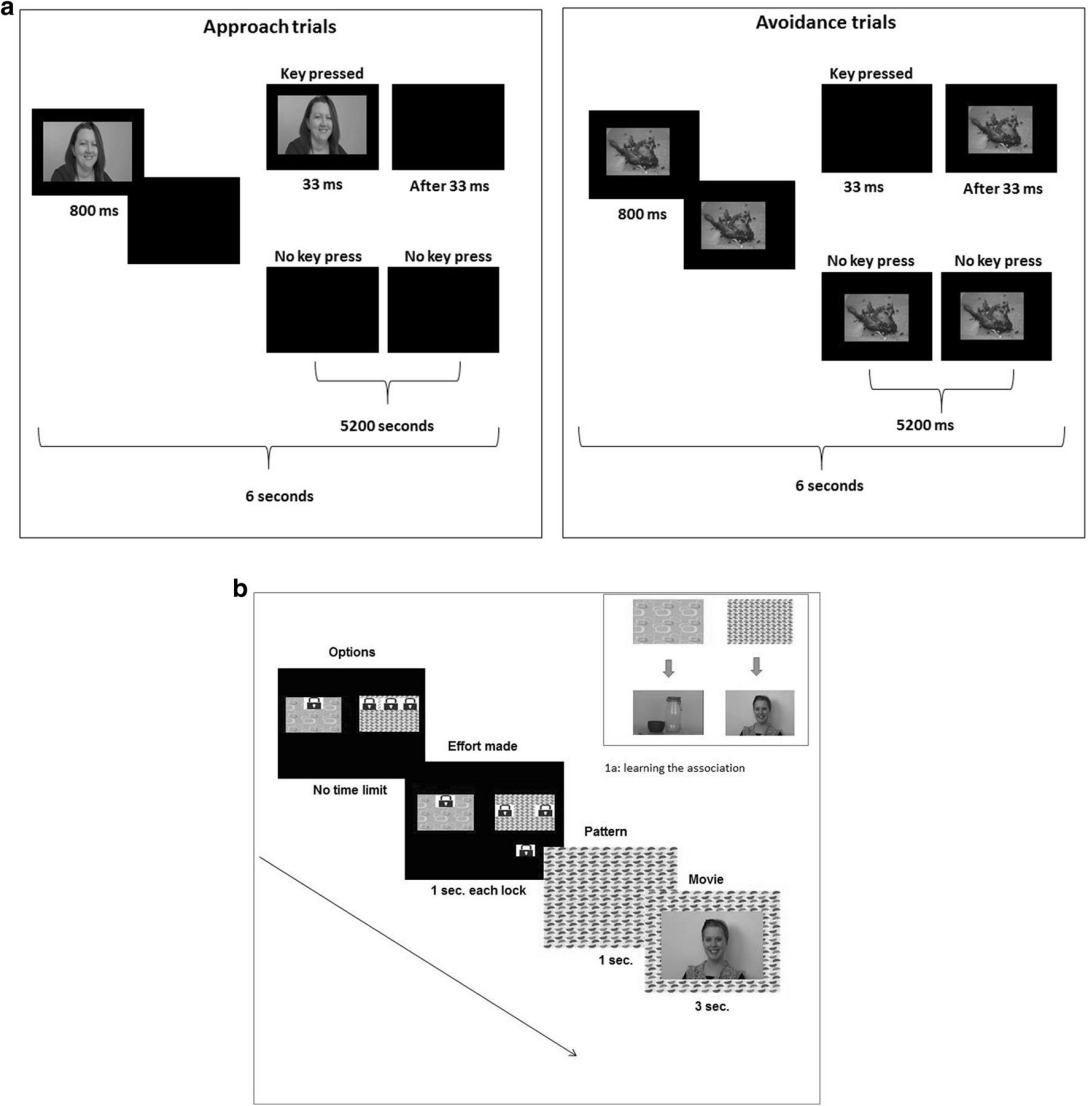
**a** Trial structure for approach–avoidance paradigm (each key-press shows/removes the image for 1 screen refresh which is 33 ms); **b** trial structure for Choose a movie paradigm. Participant is presented with two boxes (each linked with one set of stimuli e.g. pink spotty box with social movies) with different numbers of locks (1–3 on each side). They chose any one box and touched its locks to open. Each lock took about 1 s to open. When all locks on any one box were removed the box extended to full screen and one of the linked movies played for 3 s

Recently, the choose a movie (CAM) paradigm has been developed by Dubey et al. (2015, 2017) that aims to overcome these limitations. This paradigm is based on the principle that approach behaviour can emerge from the learned association between rewarding stimuli and cues, where cues then initiate the approach behaviour (Berridge 2004). Therefore, in this paradigm participants initially learn the link between coloured boxes and two types of stimuli: social and non-social and later they make choice when presented the cue and not the stimuli (see Fig. 1). CAM uses brief movies of smiling people and rotating objects as stimuli that have higher ecological validity than still images (Hanley et al. 2013). Participants are presented with the choice between two boxes each linked to one set of movies (e.g. social and non-social). These boxes are presented with different levels of effort represented by images of locks over the boxes. The number of locks can vary from 1 to 3 on each box. To open any box a participant needs to remove all the locks from it and each lock required a distinct keypress. Participants are free to choose any one box to look at the linked stimuli. So on every trial, they are encouraged to make a trade-off between their preferences for stimuli (movie) and the level of effort (key-presses) while making a decision about which box to open. Here, the decisions about stimuli are made prior to seeing them and is less likely to be influenced by the low-level features of the stimuli. The CAM paradigm assumes that while choosing which stimulus to view, participants make a trade-off between the intrinsic reward value of each item and the effort (number of key-presses) required to view it. Hence it is able to quantify the approach motivation for the target stimulus by measuring how frequently a participant made a higher effort to look at it while he/she had the low effort alternative available.

Though CAM attempts to overcome limitations of AA paradigms the two tasks have never been compared directly. This study aims to compare these two paradigms for measuring social motivation in typical adults. As it is known that typical adults have a high social motivation (Flores et al. 2015; Shore and Heerey 2011). We expect that typical adult participants will show high social motivation on both these paradigms. Both tasks will employ social (direct gaze smiling face) and non-social (regular household object) images/movies as stimuli and will be compared for their sensitivity to elicit a preference for social over non-social stimuli in typical adults. Also as CAM proposes to overcome the limitations of AA we expect that it might present clearer results than AA.

### Social motivation and autistic traits

Social difficulties are one of the core characteristics of Autism Spectrum Conditions (ASC). Any tool that aims to measure autism focuses on the social difficulties to a great extent. The widely used Autism-Spectrum Quotient-AQ (Baron-Cohen et al. 2001) also focuses on social interactions as a major factor in screening people with autism (Hoekstra et al. 2008). Chevallier et al. (2015) suggest that a disturbance in social motivation or reduced social motivation might result in social interaction difficulties seen in ASC. Although, AQ does not claim to measure social motivation as a separate dimension, it does measures the propensity to engage with other people. This means it might serve as a good tool to compare if the people who score high on this tool (and have high social difficulties) would also perform similarly (higher social difficulties) on the behavioural tools of social motivation. AA paradigms have been used previously to estimate social motivation in relation to autistic traits/ASC but they have produced conflicting findings. Silva et al. (2015) used an AA paradigm with adolescents with and without ASC and found that participants with ASC approached positive cartoon images and avoided positive real social images more than the typical controls providing evidence of social avoidance in this population. Ewing et al. (2013) using an AA paradigm with adolescent groups of ASC and matched controls found that both these groups made a high effort to see non-social stimuli. In a different study Deckers et al. (2014) used an AA paradigm along with the “wish for social interaction scale” to measure social seeking in ASC. Results showed that although participants with ASC expressed a reduced desire to have social interaction on the subjective rating scale, they approached both social and non-social stimuli equally on the AA paradigm. These findings indicate that there might be a dissociation between *reported* and *real* social seeking behaviour as measured by the AA paradigm. Overall, studies using the AA paradigm to explore social motivation in ASC have reported mixed results.

CAM paradigms have been used previously with adults with autism (Dubey et al. 2015), adolescents (Dubey et al. 2017) with autism, and independent typical groups (Dubey et al. 2015). All these studies consistently report a negative correlation between the autistic traits/autism and social motivation. CAM results show that typical people make a higher effort to look at social stimuli than non-social, however, their social seeking is influenced by the effort involved in the paradigm and on occasions they trade-off their stimuli preference for the low effort. Similarly, people with ASC or high autistic traits make more effort to look at the non-social stimuli and like typical groups, they too are influenced by the effort. However, none of the studies have directly compared CAM with other measures of social motivation. In the current study, we aim to compare CAM with AA paradigm to measure social seeking behaviour in a cohort of typical adults. We believe that a direct comparison of social seeking behaviour as evaluated by these two tools in the same sample of typical adults will allow us to examine if any of these measures has higher sensitivity to evaluate preference for social stimuli over non-social stimuli. We will also evaluate the autistic traits of the participants to see if these traits can predict their strength of preference for social stimuli. Based on the theory of reduced social motivation in autism (Chevallier et al. 2012) we expect that both AA and CAM will demonstrate similar levels of negative correlation between social seeking and autistic traits of the participants.

## Method

### Participants

Participants were recruited through the School of Psychology’s research participation scheme and posters on the university campus. To explore the relationship between social preference on the two paradigms and autistic traits we recruited participants with a wide range of autistic traits. More than 400 undergraduate students completed the online version of the of the adult autism quotient scale (AQ) (Baron-Cohen et al. 2001) (this data is also reported at James et al. 2016). Our target sample size was 48 participants for the behavioural study, based on our previous experience of using the CAM task in our lab. From the participants who completed screening, 49 undergraduate students agreed to visit the lab for the second part of the study. Data from two participants was not included in the analysis due to their inattention to the paradigm and poor adherence to the instructions. Data from all the other 47 participants (24 females) between ages 18–41 years (M = 20.06 years, SD = ± 4.45) is reported here. These participants received course credit or an inconvenience allowance for their participation. They were informed about the larger aim of the project but were not aware of the specific aim of the study until they finished all the experimental paradigms.

### Tools

#### Approach–avoidance paradigm

The paradigm was presented using MATLAB with Cogent toolbox on 12 × 6.5-inch screen of a Samsung Ultrabook. The 60 images used in the paradigm were taken from an internet search. The stimuli consisted of three types: 20 images showing an individual with a direct gaze and a social smile (10 females, 10 males); 20 images of regular household objects; 20 images of disgusting things such as animal faeces, dirty toilet, dead animal, bugs etc. The aim of using aversive images was to provide a strong contrast to the social/non-social images and ensure participants were attentive to the images being presented. All the images were free of copyright restrictions and could be used for personal/academic purposes. To control the influence of low-level features, such as bright colours, images were transformed to black and white format. The background of the images was left unaltered to make sure they looked natural.

There were two phases of the paradigm: the approach phase and the avoidance phase. For the approach phase participants were informed that they will see some pictures on the screen and followed by 6 s of a gap during which they can either look at the blank screen or bring the picture back by pressing key “H” (Fig. 1a). For the avoidance phase participants were informed that they will see some pictures on the screen for 6 s and they can remove the picture (to look at blank screen) anytime by pressing key “H”. Participants were also informed that each trial duration is fixed to 6 s and does not increase or decrease with their key-presses. Each key press brought the image back or removed it only for a single refresh (33 ms) of the screen. Participants completed 60 trials of the approach task with all 60 images in a pseudorandom order, and also 60 trials of the avoidance task with the same 60 images in a pseudorandom order. Presentation of the two phases: approach and avoidance, was randomised between participants. For both phases, the responses were recorded in milliseconds of viewing the images.

#### Choose a movie paradigm

The paradigm was presented on a touch screen 12 × 6.5-inch screen of a Samsung Ultrabook laptop using MATLAB with Cogent toolbox. Stimuli consisted of 10 short (3 s) movies of smiling adults (social stimuli) and 10 short movies of objects slowly rotating on a turn-table (non-social stimuli) (See Dubey et al. 2015 for details). Participants were first shown a pattern (e.g. orange stripy box) and were informed that this box will always be linked with one set of stimuli (e.g. smiling adults) and the other pattern (e.g. green spotted box) will always be linked with the other set of stimuli (e.g. moving objects). They were then presented with 10 learning trials in which they saw one box on the screen with locks on them and the participants were expected to touch the locks to open the box and see one of the movies from the linked set of stimuli. These trials aimed to familiarise participants with the paradigm and the kind of stimuli. After the learning trials, the participants were informed that they will see both the boxes in the next trials and they can choose to open any one of those to look at the movie they like. Participants were then presented with 60 experimental trials of choosing between the two boxes. The boxes could have 1–3 lock on each of them (Fig. 1b). Within these 60 trials, 24 trials presented 1 lock on one box and 3 locks on the other box, 12 trials presented 1 lock on one box and 2 on the other, 12 trials presented 2 locks on one box and 3 on the other, and 12 trials presented equal number of locks on both the boxes. Each lock took one touch to be removed therefore more locks meant more touches or effort. Here the participants were encouraged to make the trade-off between their preference for a movie and the effort required to look at it.

### Procedure

Informed consent was obtained from all individual participants included in the study. The participants first completed an online version of the AQ (Baron-Cohen et al. 2001). They were then invited to the lab where they completed CAM and AA paradigms. The presentation sequence of the two paradigms was counterbalanced between participants to prevent the influence of tiredness or boredom on any one of them. For the CAM paradigm, participants completed 60 trials giving a fully counterbalanced measure of preference for each of the two movie types. After the CAM data were collected the participant took part in further tasks which are not analysed here. For the AA paradigm, they completed 60 trials of approach and 60 trials of avoidance in a counterbalanced order. Participants were debriefed about the specific aim of the study at the end of the session.

## Results

### Social motivation measured by approach–avoidance paradigm

**Fig. 2:**
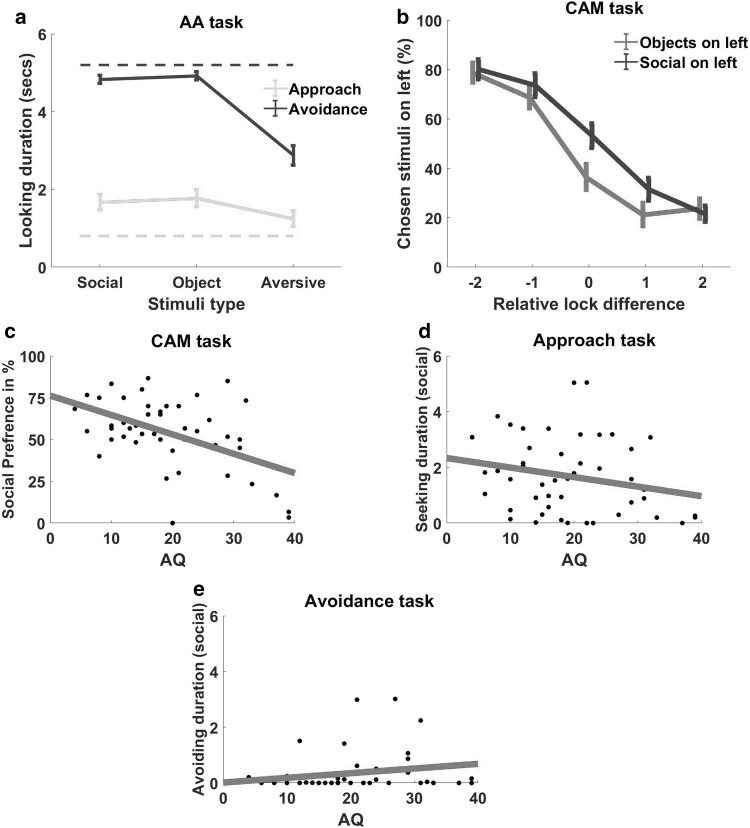
**a** Mean duration (seconds) of looking at the three sets of images in approach and avoiding sets. Upper dashed line (blue) indicates what the looking time would be for the avoidance task with no keyhits. Lower dashed line (green) indicates what the looking time would be for the approach task with no key hits; **b** preference for each stimulus (two coloured lines) over different levels of relative efforts (e.g. − 2 on X axis represents 1 lock on left and 3 on right side); **c** correlation between autistic quotient and social preference on the CAM paradigm measured as percentage of time participant chose social stimuli over non-social, irrespective of effort level; **d** correlation between autistic quotient and social seeking on AA task measured as the average duration of looking at social stimuli on the approach phase; **e** correlation between autistic quotient and social avoidance on AA task measured as the average duration of avoiding social stimuli on the avoidance phase. (Color figure online)

In the approach set of AA paradigm, each key-press made the picture available for 33 milliseconds only. Hence to look at the picture longer participant needed to make very quick regular key-presses. More frequent key-presses ensured a longer exposure to the available image. Hence the total duration of viewing an image indicates the effort made by the participant to look at it and therefore can be seen as an estimate for motivation to seek that stimulus. Similarly, in the avoidance section, each key-press removed the picture from the screen for 33 ms. The duration of looking at the images during this phase was calculated by subtracting the duration when the key was pressed (to remove the image) from the total stimulus presentation time i.e. 6 s. The looking duration was then averaged across the images for each category social, non-social and aversive. As Fig. 2a illustrates during the approach trials participants spent an average duration of 1.67 (*SD* = 1.37) seconds looking at the social images, 1.77 (*SD* = 1.48) seconds looking at the non-social images and 1.25 (*SD* = 1.36) seconds looking at the aversive images. This shows a significant difference in the looking time for the three sets of images *F*(2, 92) = 4.586, *p* = 0.013*, ηp^2^ = 0.091. The posthoc comparison between social versus aversive (*p* = 0.086), non-social versus aversive (*p* = 0.039) shows that participants spent significantly less time viewing aversive images. However, there was no significant difference between the approach duration for social versus non-social images (*p* = 1.00*)*. For the avoidance phase, participants spent an average of 4.83 (*SD* = 0.73) seconds looking at the social images, 4.92 (*SD* = 0.71) seconds looking at the non-social images, and 2.88 (*SD* = 1.73) seconds looking at the aversive images. The comparison between them suggests a significant difference in the viewing duration of these stimuli *F*(1.11, 51.089) = 57.39, *p* < 0.001*, ηp^2^ = 0.555. The posthoc comparison shows a significant difference in the duration of avoidance between social versus aversive images (*p* < 0.001), and non-social versus aversive images (*p* < 0.001), however, there was no significant difference in the duration of avoidance between social versus non-social images (*p* = 0.579).

### Social motivation measured by CAM paradigm

The CAM paradigm measures the preference for social over non-social stimuli against different levels of effort. We coded each trial in terms of whether left hand item was chosen (Choice), whether the left hand item was ‘social’ or ‘nonsocial’ (Stimuli) and the relative number of locks on the left hand item compared to the right hand item (Relative Effort). For example if left side had social box with three locks while right side had non-social box with 2 locks, and the participant chose the left side, we code this trial as “stimuli = social” and “relative effort = 1” (locks on the left side–locks on the right side). As each trial has a binary outcome, we used logistic regression model to analyse the data. In this model, we tested how the factors Stimuli and Effort predicted participant’s choices, using a logistic link function. The results showed that participants were significantly influenced by the stimuli type (Wald χ^2^ = 18.68, *p* < 0.001) i.e. they clearly showed a preference for one stimulus over the other. Their choice was also influenced by the effort levels (Wald χ^2^ = 51.07, *p* < 0.001) i.e. they did not choose any one stimuli rigidly over different effort conditions but were being careful to choose the low-effort option. There is also a significant interaction between effort and stimuli (Wald χ^2^ = 13.06, *p* < 0.011) and as Fig. 2b shows our participants preferred social stimuli on most of the effort levels but the preference was more prominent when the effort difference between the choices was zero (i.e. both the stimuli were presented with the same number of locks). This preference was also strong when the effort difference was + 1 or − 1 locks, but as the difference increases the preference for any specific stimuli decreased. This suggests that participants make a trade-off between their social preference and required effort, which can be clearly quantified using CAM paradigm.

### AQ and social motivation on two paradigms

We used *Pearson* correlation coefficient to explore the relationship between autistic traits (AQ) and social motivation as measured by “looking duration” (making key presses to expose the image for a longer duration) on the approach set of AA paradigm. Similarly relation between autistic traits and social motivation as measured by the CAM paradigm (percentage of trials on which participant chose social over no-social stimuli). We also looked at the correlation between autistic traits and social avoidance duration (key press made to suppress the exposure of images) as measured by the avoidance set of AA paradigm. The results as presented below suggest that there was no significant correlation between the duration spent on looking at social images [*r* (45) = − .224, *p* = 0.130] (Fig. 2d) or avoiding social images [*r* (45) = − .205, *p* = 0.167] (Fig. 2e) on AA paradigm and the AQ of participants. However, there was a strong negative correlation [*r* (45) = − .499, *p* < 0.0001] between AQ scores and social preference on the CAM paradigm (Fig. 2c). There was no correlation between the social seeking on the AA paradigm and CAM paradigm.

## Discussion

The aim of this study was to compare two different measures of social motivation: AA and CAM paradigms to (1) measure social motivation in typical adults and (2) see the relation between their autistic traits and social motivation. Unlike what is suggested by the literature, participants did not show a preference for social stimuli over non-social stimuli on AA paradigm. However, they showed a clear preference for social stimuli on CAM paradigm which also correlated with the autistic traits of the participants. In the following sections, we will discuss the factors on which these two measures differ and how that might have influenced the results on the different tasks.

### Theoretical foundation

Both AA and CAM paradigms originate from the theoretical premise that people make an effort to approach the stimuli that have a higher reward value for them. The AA paradigm sticks to this conceptualization strictly, however, CAM paradigm takes it a step further by including some other factors involved in incentive motivation (Berridge 2004). As per Berridge’s incentive motivation theory, the behavioural approach emerges from the presence of cues that activate anticipation of reward and this is further modulated by the subjective state of the person. In CAM the boxes present at the choice stage of the trial, serve as the cues for the social/non-social stimuli. The anticipation of looking at the stimulus with high subjective reward value (social stimuli for typical people) activates the approach behaviour but it is also modulated by the relative level of effort involved in approaching it. Hence, the participant re-evaluates the decision to approach or not on every trial.

Our results showed that a relatively simpler AA paradigm is able to discriminate the preference between neutral and aversive stimuli, which might emerge from a stronger negative valance for aversive stimuli i.e. there is a significant difference in the looking time for aversive versus social and non-social stimuli. However, this paradigm did not reveal any preference between social and non-social stimuli, perhaps because typical adults might not have any strong non-preference for any one of these. Furthermore, participants might prefer looking at any image that is not aversive than looking at a blank screen hence leaving the little difference in the seeking behaviour for social and non-social stimuli. This suggests that AA might be a useful tool to measure the strength of avoidance of aversive images, rather than the strength of approach towards positive images. On the other hand, the CAM paradigm that evaluates relative preference of two stimuli at different levels of effort is able to highlight the difference in the approach motivation for two sets of more positive stimuli. As both these paradigms measure preference for social and non-social stimuli in the same set of participants and only CAM is able to demonstrate strong preference for social stimuli, it can be said that CAM has higher sensitivity to estimate motivation for seeking social over non-social stimuli.

Our data also show a strong correlation between AQ scores and social preference on the CAM paradigm. This replicates our findings in previous studies (Dubey et al. 2015). To the extent that the AQ can be taken as a measure of social motivation, our study thus provides evidence for the concurrent validity of the CAM task, showing that CAM taps into the same ‘social motivation’ as the AQ. However, the AQ was not designed as a ‘pure’ measure of social motivation, and other studies suggest that autistic traits are not the only factor determining how much people find social interaction rewarding (Foulkes et al. 2015). Thus, further work will be needed to identify the best questionnaire measures of social motivation, real world measures of social motivation and how these relate to lab-measures like the CAM.

### Approach motivation for general category versus specific stimuli

It is known that the responses elicited by looking at a stimulus might be influenced by its low-level features rather than the learned awareness of its pleasant properties (de Bordes et al. 2013; Itier et al. 2007). On AA paradigms, the participants make the decision about approaching or not a stimulus while the stimulus is already being presented to them. Here, it may be hard to separate the approach behaviour of the participant emerging from the low-level features (brightness/colour etc.) from a more general approach motivation for a set of stimuli e.g. social, objects etc. For example, a participant might make more effort to look at an image of a person who resembles his/her mother. Here the behaviour of making a high effort for one particular social image cannot be generalised to an encompassing high social motivation. Similarly, a participant might make a high effort for some specific non-social/social image from the sample images, only because of their stylistic feature such as colour. This again might result in higher seeking value this particular image but might not necessarily reflect the high motivation to seek or avoid other stimuli from that category. This suggests that on an AA paradigm the approach behaviour of the participants might be influenced more by the features of the individual images rather than the complete category of social / non-social stimuli. The features of individual images may not influence the response of the participants in the CAM paradigm because CAM uses cues (the patterns associated with two sets of stimuli) to present the stimuli. The participant makes a decision about approaching it on the basis of the learned association between a cue and a stimulus category. Hence, on CAM the approach behaviour of the participant is more likely to reflect the motivation for the general category of the target stimuli than any specific images of stimuli.

### Relative reward value of the approached stimuli

In the present study, AA paradigm failed to show any preference difference between social and non-social stimuli. One possible explanation for this is that on each trial the participant is presented with only one stimulus on the screen and he or she is expected to press keys to approach or avoid it. Therefore, this paradigm measures the preference for each set of stimuli against looking at a blank screen (i.e. doing nothing). However, it has been suggested in the literature that ‘doing nothing’ is a negative experience for many people. People try to avoid ‘doing nothing’ by even engaging in non-rewarding activities (Wilson et al. 2014). Therefore, the choice between looking at an image or a blank screen in the AA paradigms might make participants press the key to avoid the negative experience of a blank screen rather than seeking reward. Furthermore, it is not clear if the choice between viewing an image and viewing a blank screen has much ecological validity. In real life situations, people generally have multiple options to choose from and their final choice is a result of a complex evaluation of the utility of each option against others. For example, Zellner, Allen, Henley, and Parker (2006) found people gave higher ratings for diluted juice when it was presented against water, but lower ratings when it was presented against a more concentrated juice which was sweeter. In the present study, perhaps the high approach behaviour for non-social stimuli (in absence of alternative but looking at the blank screen and doing nothing) observed on AA paradigm, might have emerged from these two factors. And unfortunately, it is hard to know the extent to which these factors might have influenced the approach behaviour of each participant.

CAM paradigm presents two stimuli to choose from, hence it measures the relative reward value of the stimulus under investigation. This kind of reward value of a stimulus may be more predictive of real life behaviour where choices are made in relation to each other. However, CAM paradigm presents a choice between only two stimuli whereas in real life situations people may have more than two available options and they make a complex comparison of the utility of all these options before making a decision. It will be interesting to see if the preference for social stimuli as observed on CAM in this study would remain the same if there were more than two choices.

### The ecological validity of the stimuli

In the AA task the stimuli is presented as the still images, that were not rated for valance or arousal hence we cannot be sure if the low level features or the significant difference in the valance of the images in each category might have any influence on the results. On the other hand, CAM paradigm uses short videos of stimuli such as a person making eye contact and smiling, or objects rotating. Although, we do not have valance ratings for the social and non-social stimuli used in this task as well however, it is shown that the dynamic stimuli have higher ecological validity than the still images (Hanley et al. 2013). Therefore, they are more likely to elicit typical behaviour of the participant than still images or line drawings. Perhaps the closer simulation of natural social stimuli through movies might enhance the experience of hedonic pleasure from them than the still images. This might be another reason why we observed a higher preference for social stimuli on the CAM paradigm than the AA paradigm. Unfortunately, due to the very nature of the AA paradigm, it is not feasible to use video stimuli in it.

### Task performance in relation to autistic traits

As it is accepted that the social difficulties form an essential component of ASC and also that people with ASC might have lower social motivation (Chevallier et al. 2012), we here compared the CAM and the AA tasks with the well-established tool—AQ—to measure autistic traits in general population. We expected that people exhibiting higher level of social difficulties on AQ would also show lower social motivation on the two behavioural tools hence suggesting higher concurrent validity of the measures. However, the comparison between the social preferences on the two paradigms in relation to autistic traits showed that on the AA paradigm participants’ effort to look at the social images has little association with their autistic traits, but on the CAM paradigm, this association is strong. These findings are similar to what has been reported in the literature earlier. Ewing et al. (2013) did not find any group difference between ASD and non-ASD adolescents when using a variation of the AA paradigm to measure social motivation. On the other hand, Dubey et al. (2015) showed a clear difference in the social motivation of adults with and without ASD when using the CAM paradigm.

This difference might be attributed to two main features of these paradigms. Firstly, the CAM paradigm presents the participant an option between two stimuli to choose from. As suggested by Sasson, Turner-Brown et al. (2008) the preference for social stimuli in ASD is strongly influenced by the other stimuli competing for attention. People with ASD are more likely to explore social stimuli if they are presented with low autism interest objects than when they are presented with high autism interest objects such as trains. Therefore the social and non-social preference without any competing stimuli as measured in AA paradigms might have little relation to the autistic traits of the person, while the preference for one over the other as measured in CAM might evoke a relative preference that is closely linked to the autistic traits of people. A second reason why a social preference may not have been observed on the AA paradigm is because the stimuli had lower ecological validity than the CAM paradigm. It has been suggested that the atypicality of visual attention in ASD becomes more prominent as the ecological validity of the stimuli increases (Chevallier et al. 2015; Hanley et al. 2013).

### Limitations

For every psychological study, there are a number of possible tasks which can be used each with a number of different parameters. Here, we used one particular version of an approach–avoidance task and one particular version of the CAM task to measure social motivation. It is important to note that our results may not generalise to all other task parameters. For example, if the AA task were modified to use dynamic stimuli, or if different images with different levels of arousal were used, then our results might not be the same. Similarly, the way “effort” is conceptualised in these two versions of the tasks is a parameter under the experimenter’s control and we picked one particular effort requirement. If we had setup the CAM task to have 10 key presses rather than 3 in high effort condition or had set up the AA to require fewer keyhits to change the screen, again the results might not be the same. Hence, we are aware that our conclusions about the AA task and the CAM task apply only to the specific versions we have tested, and not to all possible implementations of these tasks. Our inference about utility of CAM or AA tasks derived from this study cannot be generalised to the other versions of these paradigms.

## Conclusion

This study indicates that though the AA paradigm and CAM paradigm have both been designed to measure ‘social seeking’, differences in their presentation of stimuli (alternative vs. no alternative) and the nature of stimuli (images vs movies) might influence participants’ behaviour significantly resulting in a difference in the findings obtained on them. The results in relation to autistic traits suggest that CAM paradigm might be more sensitive at identifying the behavioural difference in social motivation than AA paradigm.
